# Supplementary material for: ZmASR3 from the Maize ASR Gene Family Positively Regulates Drought Tolerance in Transgenic Arabidopsis
Source: Int J Mol Sci. 2019 May 8;20(9):2278. doi: 10.3390/ijms20092278 (PMC6539908; doi:10.3390/ijms20092278)
Supplement: Supplementary file 1 [file ijms-20-02278-s001.pdf]

Table S1. The molecular features of *ASR* genes.

Gene name	Gene identifier	ORF(aa)	MW(Da)	pI	chromosome	Chromosomal localization	
						Star	End
ZmASR1	GRMZM2G136910_T01	417	15542.85	5.89	Zm10	8852553	8853933
ZmASR2	GRMZM5G854138_T02	396	14904.22	6.15	Zm2	139700113	139701207
ZmASR3	GRMZM2G044132_T02	1020	35955.05	9.23	Zm2	54473360	54474810
ZmASR4	GRMZM2G168552_T01	546	20428.94	6.3	Zm8	156640234	156641211
ZmASR5	GRMZM2G052100_T01	315	11789.99	6.65	Zm10	116077391	116078438
ZmASR6	GRMZM2G057841_T01	321	11541.82	8.05	Zm5	171162247	171162872
ZmASR7	GRMZM2G014797_T01	321	12077.77	10.56	Zm3	65662114	65662673
ZmASR8	GRMZM2G314075_T01	309	11460.85	9.66	Zm3	65665421	65666059
ZmASR9	GRMZM2G383699_T01	309	11412.81	9.78	Zm3	65739867	65740452
OsASR1	LOC_Os01g72910.1	318	11676.21	9.66	Os1	42282832	42283632
OsASR2	LOC_Os01g73250.1	549	20439.9	6.59	Os1	42460555	42462334
OsASR3	LOC_Os01g72900.1	291	10573.92	9.62	Os1	42281272	42281973
OsASR4	LOC_Os02g33820.1	318	11676.94	6.76	Os2	20165589	20166668
OsASR5	LOC_Os11g06720.1	417	15464.86	6.2	Os11	3278435	3279425
OsASR6	LOC_Os04g34600.1	690	24463.48	9.49	Os4	20934819	20936052
SbASR1	Sobic.008G049200.2	438	16274.65	5.82	Sb8	4848187	4849152
SbASR2	Sobic.006G078500.1	810	28483.34	4.92	Sb6	44420519	44422060
SbASR3	Sobic.005G050200.1	405	15172.49	6.11	Sb5	4770080	4771218
SbASR4	Sobic.006G078400.1	306	11545.73	6.65	Sb6	44407316	44408052
SbASR5	Sobic.003G158600.1	306	11269.64	9.78	Sb3	18206542	18206847
SbASR6	Sobic.003G158500.1	306	11865.37	10.19	Sb3	18201190	18203759
SbASR7	Sobic.003G437800.1	561	20584.15	6.21	Sb3	73812025	73813272
SbASR8	Sobic.004G171000.1	324	11911.2	6.98	Sb4	52358175	52358919

Table S2. sequences in this study.

Use	Primer Name	Forward Primer (5'-3')	Reverse Primer (5'-3')
1301a amplification	ZmASR1	CGGGATCC ATGGCGGAGGAGAAGCA	GCGTCGACTCAGCCGAAGAAGTGGTG
	ZmASR2	CGGGATCCATGTCGGAGGAGAAGCACCAC	GCGTCGACTCAGCCGAAGAGGTGGTG
	ZmASR3	CGGGATCCATGGCGGACGAGTACGG	GCGTCGACCTACGTACGCAAGTAGATCGATC
	ZmASR4	CGGGATCCATGGCCGACTACTACCACG	GCGTCGACTCAGTCGCAGTAGTAGGAGTAGC
	ZmASR5	GCTCTAGAATGTCTGAGGAGAAGCACCAC	AACTGCAGTCAGTTGTGGGCGTGCTT
	ZmASR6	CGGGATCCATGGCTGAGGAGAAGAAGCAC	GCGTCGACTCAGCTCTGGCCCTGCTT
	ZmASR7	CGGGATCCATGGCGCATCACTTCCTC	GCGTCGACTCAGTAACTAGCTAGTGGTGGTGTC
	ZmASR8	CGGGATCCATGGCGCATCACTTTCTCG	GCGTCGACCTAGTGGTGATGGTGTCCATGGT
	ZmASR9	CGGGATCCATGGCGCACCACTTCCT	GCGTCGACCTAGTGGTGATGGTGTCCACG

	ZmASR1	GGACTAGTATGGCGGAGGAGAAGCA	CGGGATCCGCCGAAGAAGTGGTGCT
	ZmASR2	GGACTAGTATGTCGGAGGAGAAGCACCAC	CGGGATCCGCCGAAGAGGTGGTGGTGC
	ZmASR3	GGACTAGTATGGCGGACGAGTACGG	CGGGATCCCGTACGCAAGTAGATCGATCG
	ZmASR4	GGACTAGTATGGCCGACTACTACCACG	CGGGATCCGTCGCAGTAGTAGGAGTAGCC
1305-GFP	ZmASR5	GGACTAGTATGTCTGAGGAGAAGCACCAC	CGGGATCCGTTGTGGGCGTGCTTCTT
amplification	ZmASR6	GGACTAGTATGGCTGAGGAGAAGAAGCAC	CGGGATCCGCTCTGGCCCTGCTTCTT
	ZmASR7	GGACTAGTATGGCGCATCACTTCCTC	CGGGATCCGTAACTAGCTAGTGGTGGTGTCC
	ZmASR8	GGACTAGTATGGCGCATCACTTTCTCG	CGGGATCCGTGGTGATGGTGTCCATGG
	ZmASR9	GGACTAGTATGGCGCACCACTTCCT	CGGGATCCGTGGTGATGGTGTCCACGG
	ZmASR1	CGGAATTCATGGCGGAGGAGAAGCA	CGGGATCCTCAGCCGAAGAAGTGGTG
	ZmASR2	CGGAATTCATGTCGGAGGAGAAGCACCAC	CGGGATCCTCAGCCGAAGAGGTGGTG
	ZmASR3	CGGAATTCATGGCGGACGAGTACGG	CGGGATCCCTACGTACGCAAGTAGATCGATC
	ZmASR4	CGGAATTCATGGCCGACTACTACCACG	CGGGATCCTCAGTCGCAGTAGTAGGAGTAGC
Yeast	ZmASR5	CGGAATTCATGTCTGAGGAGAAGCACCAC	CGGGATCCTCAGTTGTGGGCGTGCTT
Two hybrid	ZmASR6	CGGAATTCATGGCTGAGGAGAAGAAGCAC	CGGGATCCTCAGCTCTGGCCCTGCTT
	ZmASR7	CGGAATTCATGGCGCATCACTTCCTC	CGGGATCCTCAGTAACTAGCTAGTGGTGGTGTC
	ZmASR8	CGGAATTCATGGCGCATCACTTTCTCG	CGGGATCCCTAGTGGTGATGGTGTCCATGGT
	ZmASR9	CGGAATTCATGGCGCACCACTTCCT	CGGGATCCCTAGTGGTGATGGTGTCCACG
	ZmASR1	GCACTCTACGAGAAGCACGA	GCCTCCTTGTGGTCCTTCTT
	ZmASR2	GCACTCTACGAGAAGCACGA	TCGTGGTGCTCGTGGAATAC
	ZmASR3	ACAGCGGGTACAACAGATCG	GCTCGTTCATCAGTTCGTG C
	ZmASR4	CCTTTGCTCTGTACGAGGGG	TAGTAGGAGTAGCCGTGCCC
qRT-PCR	ZmASR5	GAAGCACCACAAGCACATGG	GTTCTCCGGGTCCTTCTTGG
	ZmASR6	AGAAGAAGCACCACCACCTG	CTTCTTCTGGTGGTGCTCGT
	ZmASR7	CCATCACTGCAGGAGCCTAC	TGGCGTCTTTCTTCTGGTGG
	ZmASR8	CGCACTCGCACAAGATCAAG	GGTGCTTCTTGGCGTCTTTC
	ZmASR9	GCCTACGCTATGCACGAGAA	TCTGGTGATGCTCGTGGAAG
	AtLTP3	GGCTTTCGCTTTGAGGTTCTTC	ATGCTAACACCGCACTTTCCAG
	AtSOS1	TCATCATCCTCACAATGGCTCTAA	ACCAACTTGCGTGGGACAACTTTA
drought-related	Rd29B	AGAGGTGGTGTAACGGGTAA	GGCTCAATGGGTTTGGTG
genes	AtCAT3	GCGTTGAAACCTAACCCGAAAA	AAACCCTCCATGTGCCTGTAATCTT
	AtSOD1	TGAGGGTGTTACGGGGACTATCT	GTGAAGGTGGCAGTTCCATCATCTC
	NCED3	CAATCATCAAACTCTCCCGCC	TCTCGTGGCTGACAAGGAAAC
Use	Primer Name	Forward Primer (5'-3')	Reverse Primer (5'-3')
	SnRK2.6	ATGGATCGACCAGCAGTGAG	GCCAATATCCTTGACGAGTT
drought-related	DREB2A	GAGGACCAGAGAATAGCCGATG	GTACTCGTAACCTCAGACGCAT
genes	COR15A	CCACCGACTCCTCTCTGCTT	AAGGGAGATTCCGAGATATGAAGA
	Atactin	GTCGTACAACCGGTATTGTG	GAGCTGGTCTTTGAGGTTTC
	Zmactin	GGGATTGCCGATCGTATGAG	GAGCCACCGATCCAGACACT

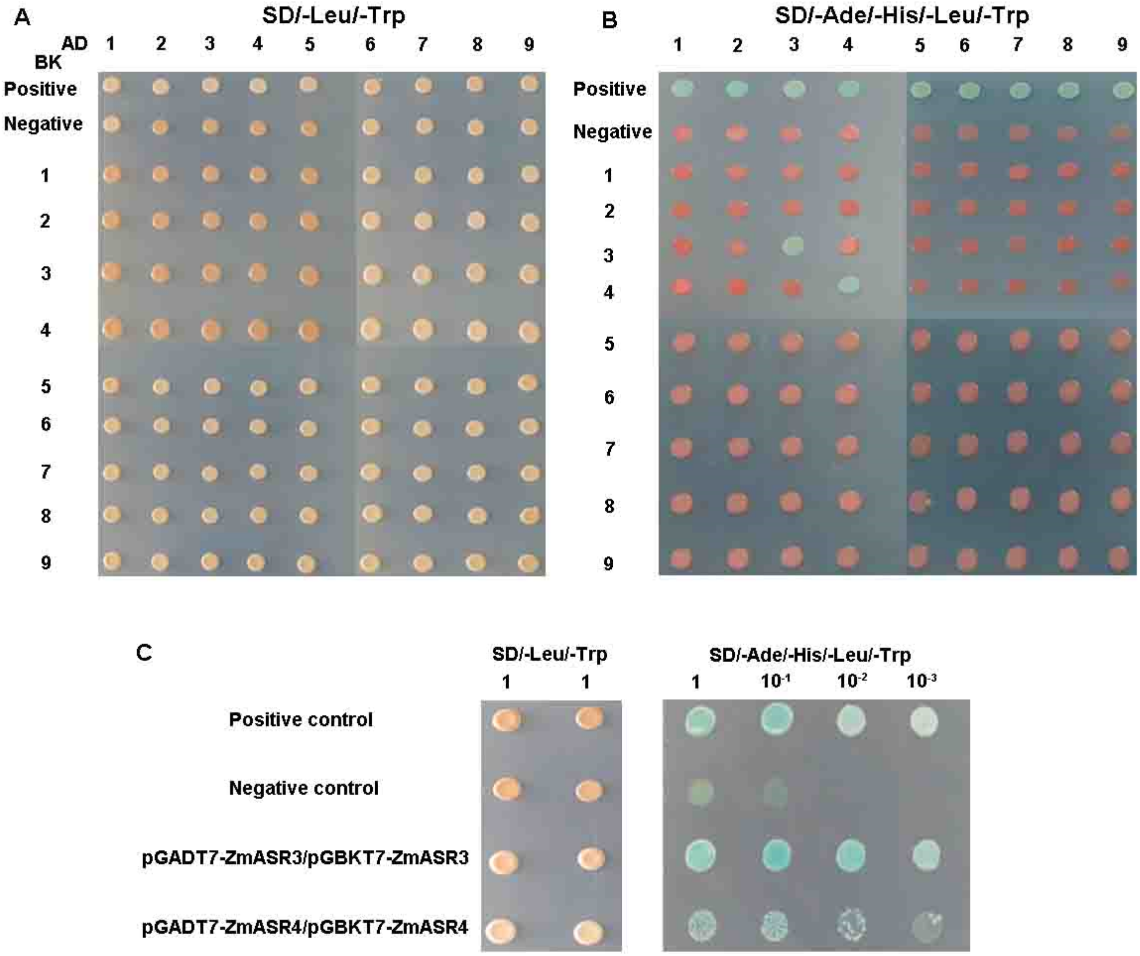

Figure S1. ZmASR3 and ZmASR4 can form the homodimer in yeast. Positive control: pGBKT7-53 + pGADT7-T; Negative control: pGBKT7 + pGADT7. 1: ZmASR1, 2: ZmASR2, 3: ZmASR3, 4: ZmASR4, 5: ZmASR5, 6: ZmASR6, 7: ZmASR7, 8: ZmASR8, 9: ZmASR9.

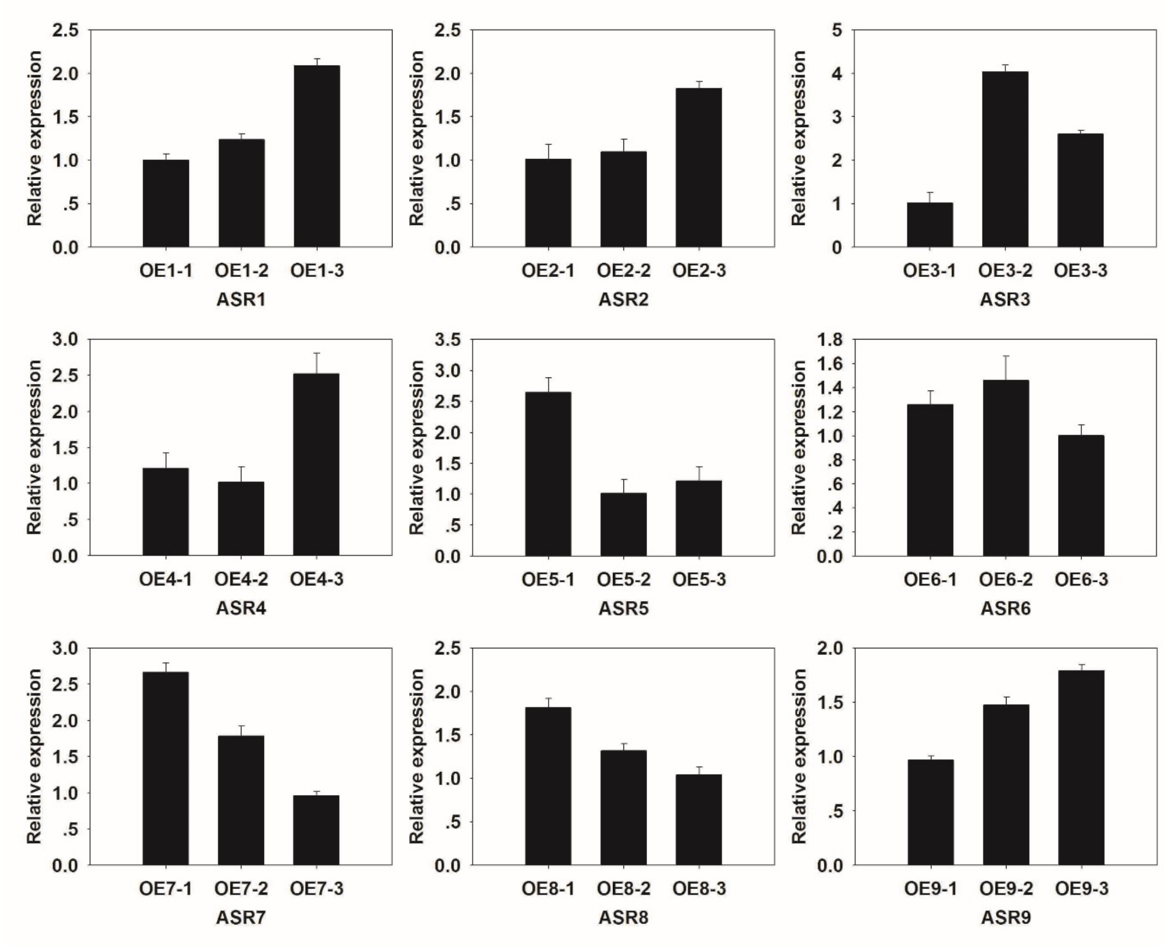

Figure S2. Expression analysis of *ZmASRs* in transgenic *Arabidopsis* assessed by qRT-PCR.

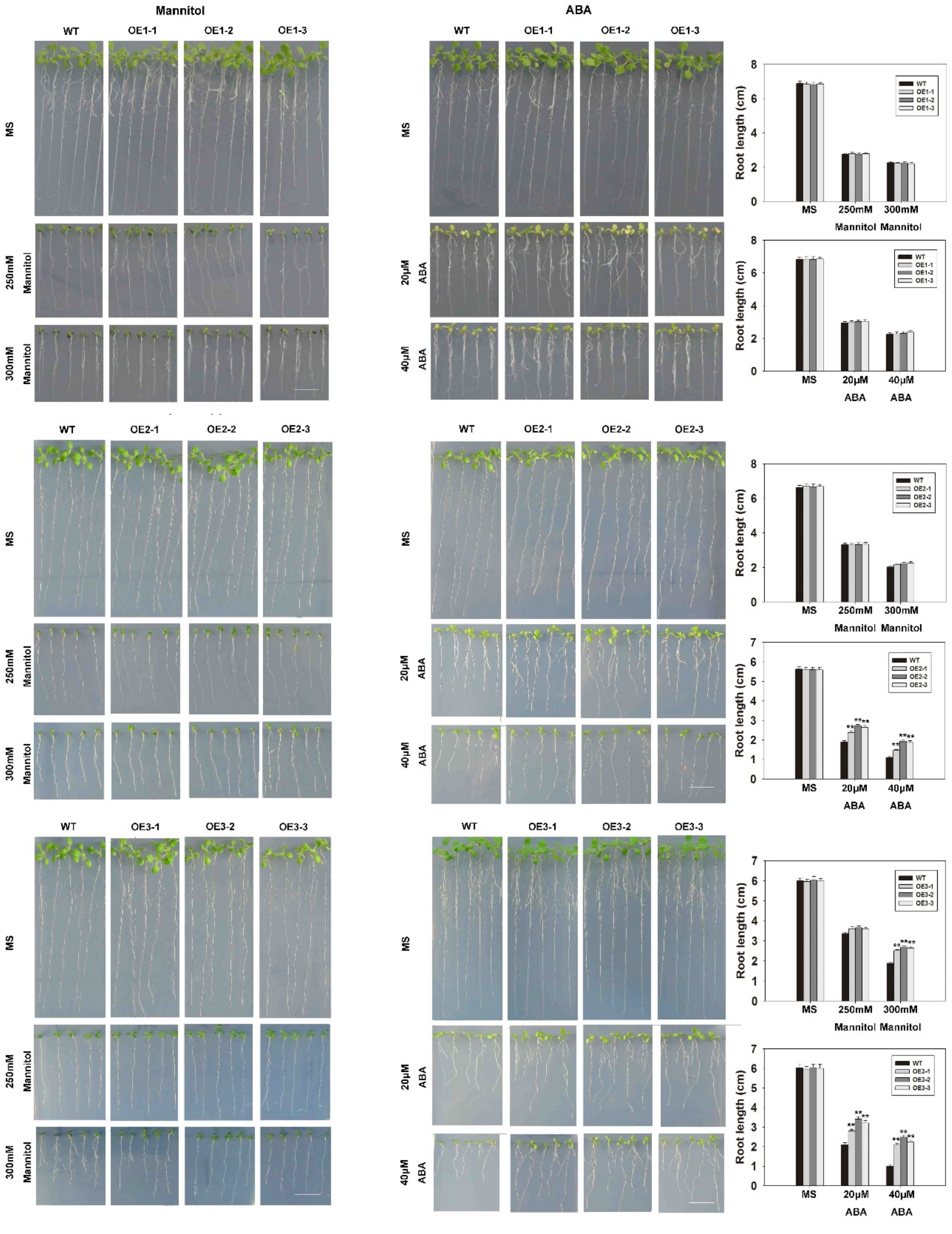
Mannitol
ABA
WT
OE1-1
OE1-2
OE1-3
OE2-1
OE2-2
OE2-3
OE3-1
OE3-2
OE3-3
MS
250mM Mannitol
300mM Mannitol
20μM ABA
40μM ABA
Root length (cm)
Root lengt (cm)
250mM
300mM
Mannitol
20μM
40μM
ABA
**

A

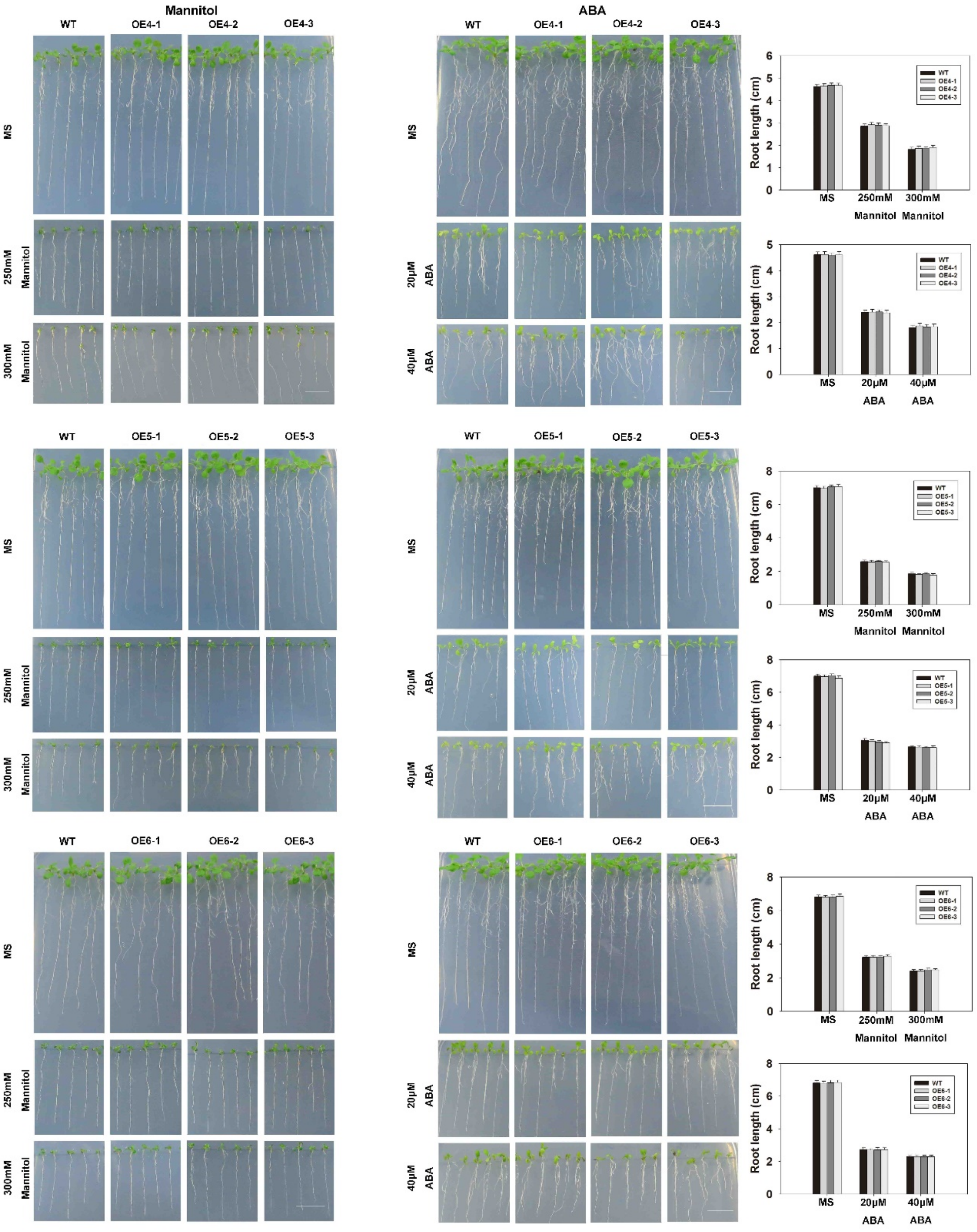
Mannitol
ABA
WT
OE4-1
OE4-2
OE4-3
MS
250mM Mannitol
300mM Mannitol
20μM ABA
40μM ABA
Root length (cm)
OE5-1
OE5-2
OE5-3
OE6-1
OE6-2
OE6-3

B

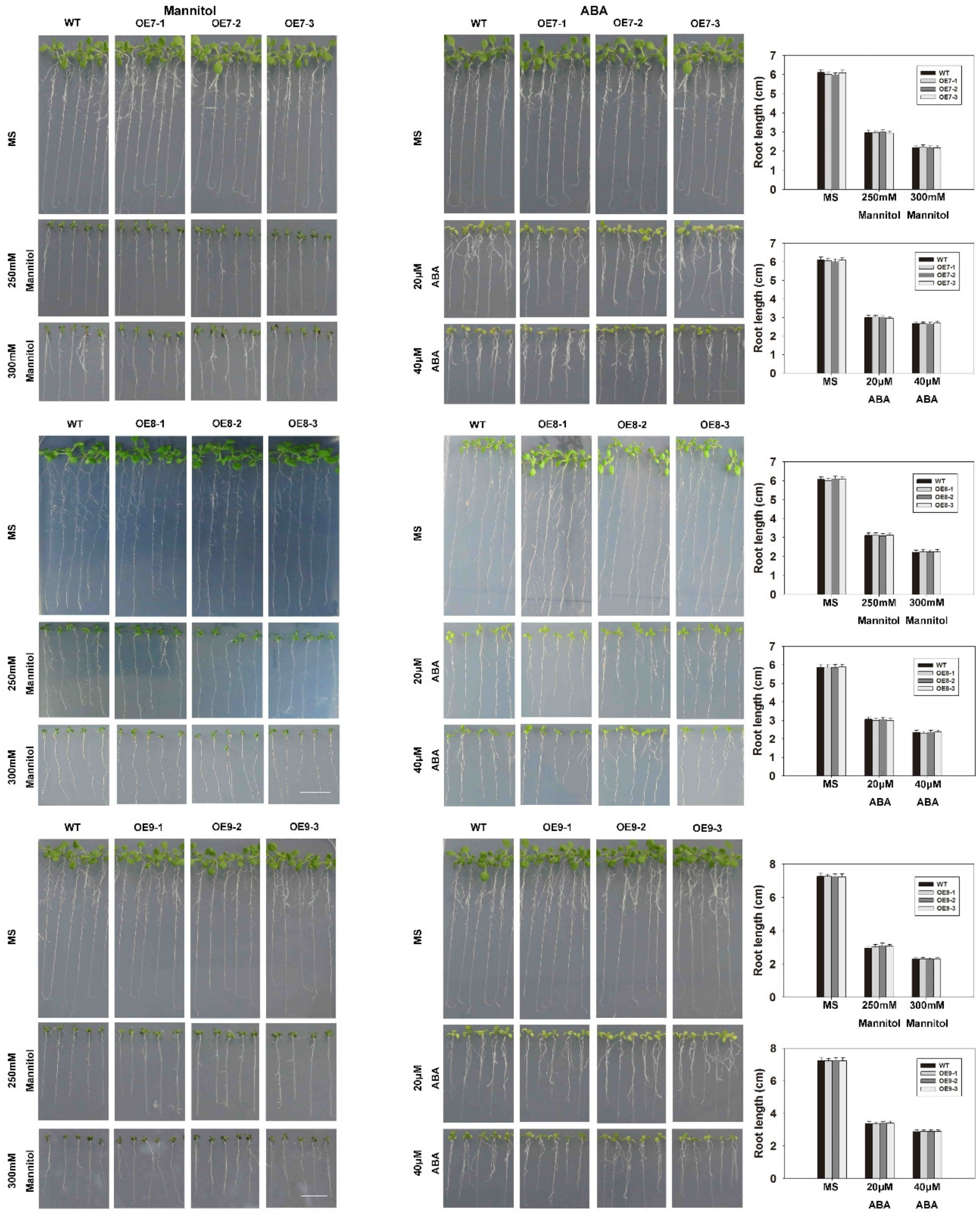

C

Figure S3. Photographs and measurements of root length of WT and *ZmASRs* transgenic seedlings at the postgermination stage on 0.5 × MS with different mannitol and ABA. Data shown represent the means (±SE) of three independent experiments (each with 50 seeds for each line). ***p* < 0.01 by Student's test. **A**: *ZmASR1*, *ZmASR2* and *ZmASR3* overexpressing lines. **B**: *ZmASR4*, *ZmASR5* and *ZmASR6* overexpressing lines. **C**: *ZmASR7*, *ZmASR8* and *ZmASR9* overexpressing lines.

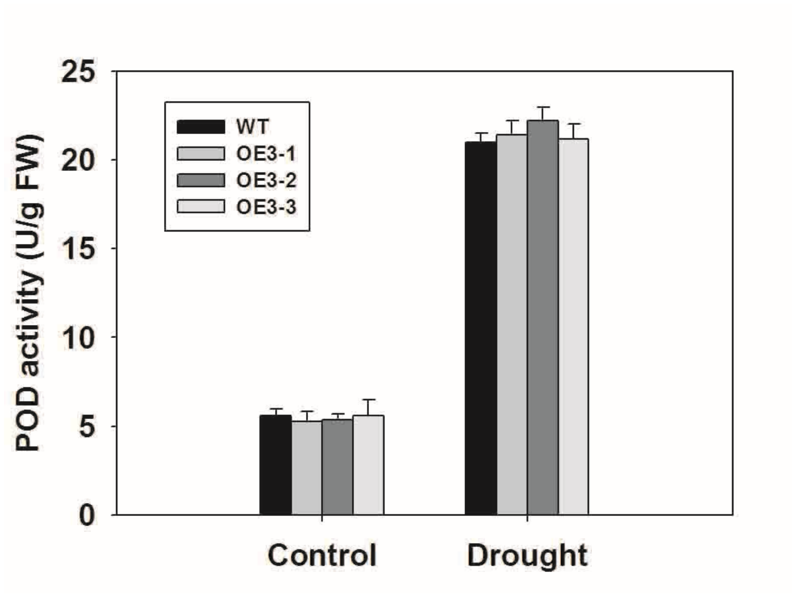

Figure S4. Analysis of POD activity of the three *ZmASR3* transgenic lines and WT plants. All samples were measured three independent experiments.

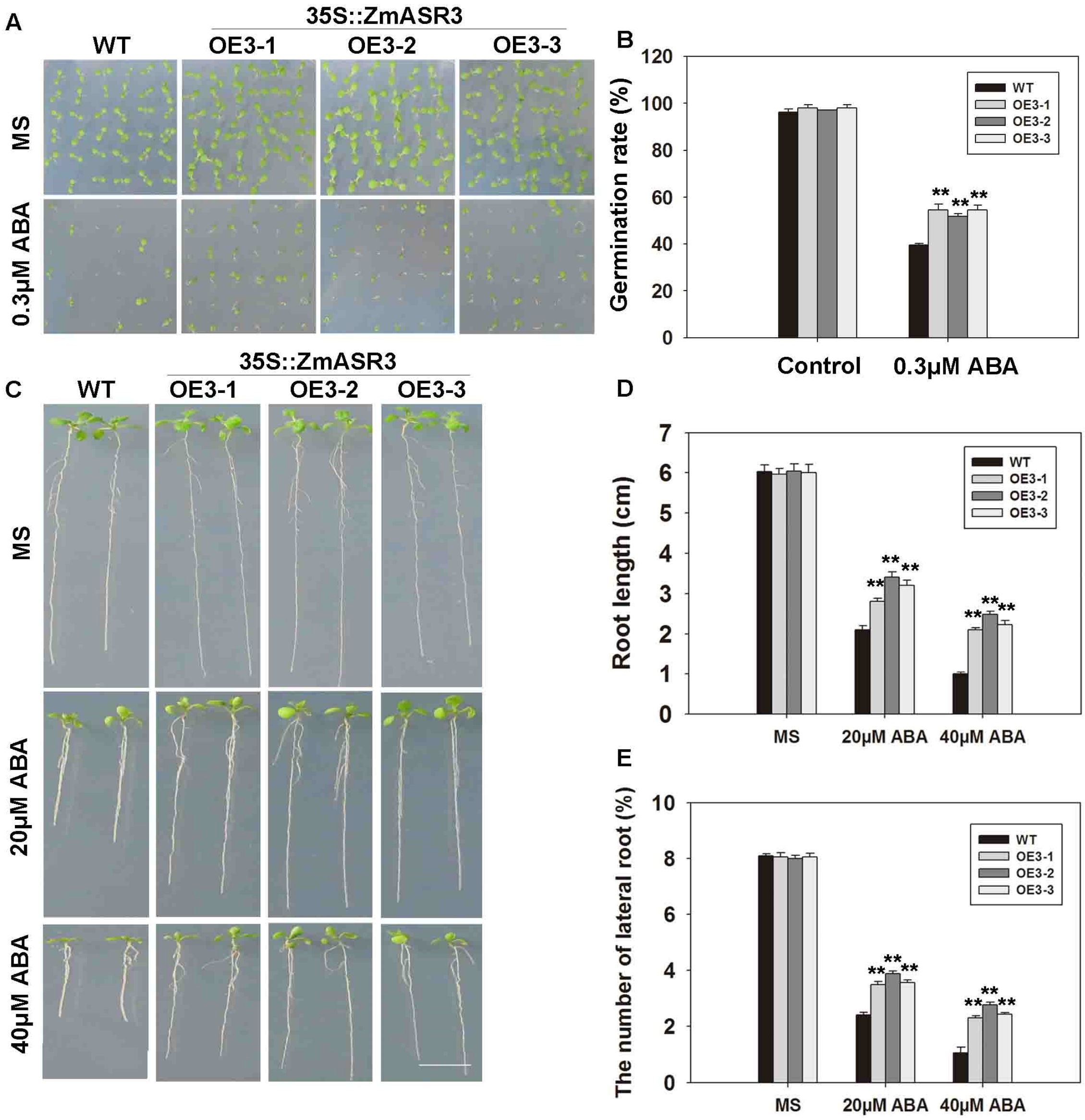

Figure S5. ABA sensitivity analysis in *ZmASR3* transgenic and WT plants. (**A**) Photographs and (**B**) quantitative evaluation of germination rate at the germination stage of WT and three transgenic lines on 0.5 × MS, or 0.5 × MS supplemented with 0.3 μM ABA. Photographs (**C**) and measurements of root length (**D**) and lateral root number (**E**) of WT and transgenic seedlings at the postgermination stage on 0.5 × MS with 20 μM or 40 μM ABA. Data shown represent the means (±SE) of three independent experiments (each with 100 seeds for each line). ** $p < 0.01$ by Student's test. Scale bar = 1 cm.

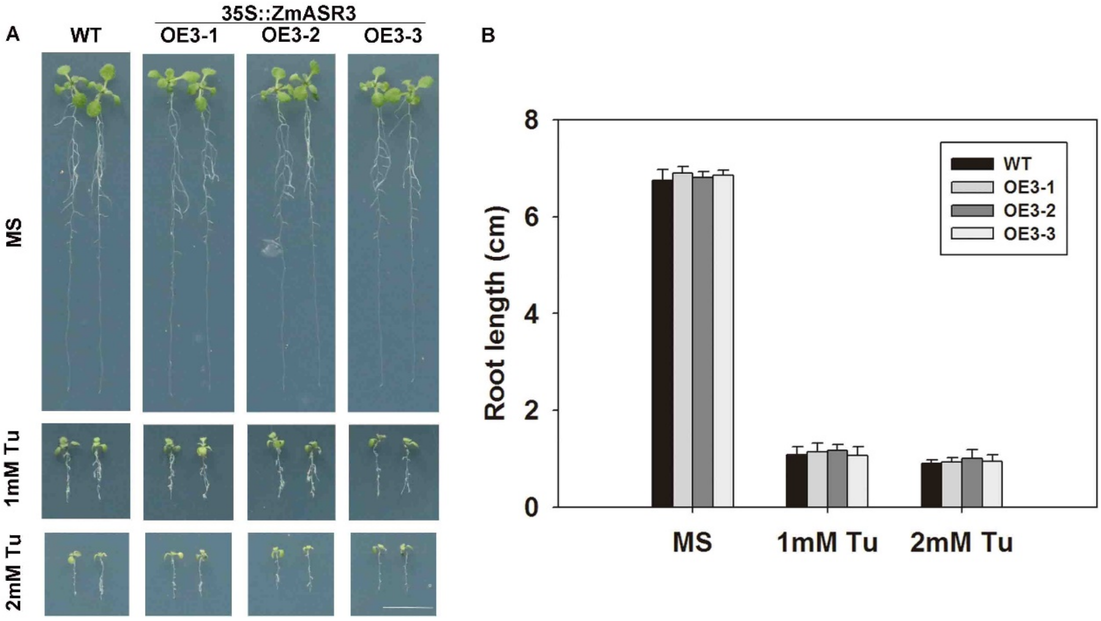

Figure S6. Effects of Tu in *ZmASR3* overexpressing transgenic lines and WT plants**.** Photographs (**A**) and measurements of root length (**B**) of WT and transgenic seedlings at the postgermination stage on 0.5 × MS with 1 mM or 2 mM Tu. All samples were measured in triplicate.
